# Elastic Hairy Nanoparticle Separator Coating for Enhanced Interfacial Stability in Lithium–Metal Batteries

**DOI:** 10.1021/acsapm.5c02697

**Published:** 2025-09-24

**Authors:** Verena Kempkes, Sipei Li, Jay F. Whitacre, Krzysztof Matyjaszewski

**Affiliations:** † Department of Chemistry, Carnegie Mellon University, 4400 Fifth Avenue, Pittsburgh, Pennsylvania 15213, United States; ‡ Department of Materials Science and Engineering, 6612 Carnegie Mellon University, 5000 Forbes Avenue, Pittsburgh, Pennsylvania 15213, United States

**Keywords:** lithium metal batteries, hairy nanoparticles, artificial solid electrolyte interfaces, separator coatings, atom transfer radical polymerization, dendrite suppression

## Abstract

Hairy nanoparticles (HNPs) provide excellent protection against dendrite formation when applied as an artificial solid electrolyte interface (aSEI) in lithium-metal batteries. ASEIs can be applied to the lithium anode in three different ways: by drop casting, electrospinning, and dip coating. However, each of these application techniques require a significant amount of handling time for the highly reactive lithium. Safer conditions during cell assembly would greatly improve the commercial applicability of lithium metal batteries. Hence, alternative processing of the HNPs in the cells was explored. HNPs with high elasticity were spray-coated on the anode side of the separator, resulting in no additional lithium handling time other than during cell assembly. This improved the common downfall of separator coatings with limited contact between the protective layer and the anode. Due to the more uniform HNP deposition, the separator coating showed a capacity retention of ∼86% after 500 cycles.

## Graphical abstract

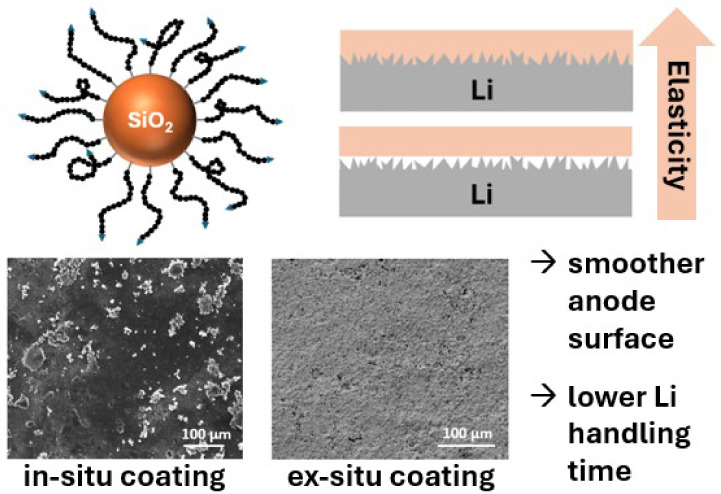

## Introduction

Secondary lithium metal batteries have great potential for future commercial energy storage systems due to their higher energy density compared to the commonly used lithium-ion batteries. However, some challenges significantly hamper the commercial use of these systems. During the charge and discharge cycles, dendrites can grow due to localized lithium deposition caused by the instability of the anode interface.^1,2^ These growing dendrites can penetrate the separator and subsequently cause a short circuit in the battery cell.^3^ Moreover, these nonuniform growths can break off and separate from the lithium anode. With the formation of the natural solid electrolyte interface (SEI), the broken-off particles can no longer be connected to the electrode. This so-called “dead” lithium, therefore, decreases the energy capacity of the cells during the cycling process.^4,5^ Various strategies have been explored during the past few years to address these challenges. Inorganic layers on the lithium anode surface have been studied.^6–8^ However, the most common challenge is their high brittleness, which leads to the formation of cracks that further exacerbate the aforementioned challenges.^9^

Organic aSEIs – such as polymers – are promising candidates for solid materials to provide flexible stabilization of the anode surface and, therefore, protection against lithium dendrites.^10,11^ The large number of available monomers, polymerization techniques, and controlled macromolecular architectures allows for a high diversity of synthesized materials.^10,12^ In addition to sufficient ionic conductivity, the materials should provide adequate mechanical stability to prevent nonuniform lithium deposition.^13^ Two polymer-based strategies to address these challenges are the application of solid polymer electrolytes as well as artificial SEIs (aSEIs).^14^ Solid polymer electrolytes provide access to all-solid-state lithium–metal batteries.^15^ However, the search for materials that can provide the required balance between ionic conductivity and mechanical strength remains challenging. ASEIs are protective layers with thicknesses significantly lower than those of SPEs.^16–18^ Since ionic conductance depends on the electrolyte thickness, this would decrease the significance of this parameter and open opportunities for more materials to be used. Due to their high ionic conductivity, poly­(ethylene oxide)-based materials have been explored as aSEIs.^16,19^ However, their low mechanical strength typically requires additives to efficiently prevent lithium dendrite formation. Grafting these polymers onto silica nanoparticles provides an anchor point for the polymer chains and, therefore, increases their mechanical properties.^20^ These so-called hairy nanoparticles (HNPs) have been used as aSEIs in lithium–metal batteries and show enhanced stability during cycling.^21,22^

Polymer-based aSEIs are generally applied to the anode directly via dip coating, electrospinning, or drop casting. However, these application techniques require a long handling time with a highly reactive anode material. Due to compromised safety during cell assembly, the utilization of these protective layers in industrial settings is impractical. By applying the protective layer to the separator, the handling time is reduced solely to the duration of battery assembly. However, these ex-situ applications typically result in reduced interfacial contact and, consequently, decreased performance (Figure 1a).^15,23,24^

**Figure 1:**
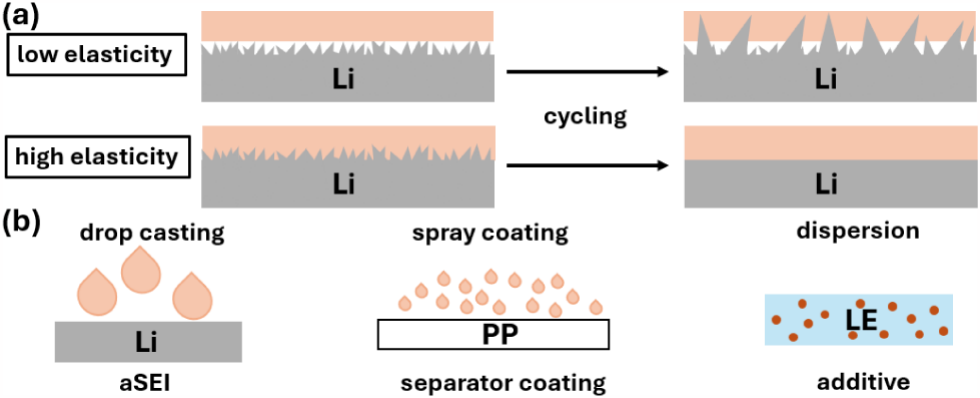
Schematic illustration of low vs high elasticity material as an ex-situ applied separator coating (a) and HNP application techniques for aSEI, separator coating, and LE additive (b).

To address this challenge, HNP materials with high elasticity were synthesized and applied as an aSEI, separator coating, and liquid electrolyte (LE) additive to lithium–metal batteries (Figure 1b). Entanglements of polymer chains are an essential part of mechanical strength as well as elasticity.^25^ To test the effect of potential entanglements, polymethacrylates with oligo­(ethylene oxide) side chains of various molecular weights were grafted from densely functionalized silica nanoparticles. They were prepared via atom transfer radical polymerization (ATRP) to ensure controlled polymer growth, leading to uniform polymer size.^26^

## Experimental Section

### Materials

Silica nanoparticles (average core diameter d = 15.8 nm, 30 wt % dispersion in methyl ethyl ketone) were donated by Nissan Chemical and used as received. 3-(Chlorodimethylsilyl)­propyl α-bromoisobutyrate was synthesized via the published route.^27^ Oligo­(ethylene oxide) methyl ether methacrylate with an average molecular mass of 500 g mol^–1^ (OEOMA_500_, Sigma-Aldrich) was purified by passing it through basic alumina. Copper bromide (CuBr_2_, Sigma-Aldrich, 99.9%), tris­(2-dimethylaminoethyl)­amine (Me_6_TREN, Ambeed, 99%), tin­(II) 2-ethylhexanoate (Sn­(EH)_2_, Sigma-Aldrich, 95%), 48 wt % aqueous hydrofluoric acid (HF, 99.99%, Sigma-Aldrich), alumina (basic, Super I, 50–200 μm, Sorbtech), lithium bis­(trifluoromethane)­sulfonimide (LiTFSI, Sigma-Aldrich, anhydrous, 99.99%), lithium nitrate (LiNO_3_, 99.99%, Sigma-Aldrich), anisole (Thermo Fisher, 99%), *N*,*N*-dimethylformamide (DMF, Thermo Fisher, 99.8%), hexane (Thermo Fisher, 98.5%), methanol (MeOH, Thermo Fisher, 99.8%), tetrahydrofuran (THF, Sigma-Aldrich, 99.5%), 1,3-dioxolane (DOL, Sigma-Aldrich, anhydrous, 99.5%), 1,2-dimethoxyethane (DME, Sigma-Aldrich, anhydrous, 99.5%), and 1-methyl-2-pyrrolidinone (NMP, Sigma-Aldrich, anhydrous, 99.5%) were used as received.

### HNP Synthesis

Silica nanoparticles were functionalized with 3-(chlorodimethylsilyl)­propyl α-bromoisobutyrate as previously reported.^27^ The silica nanoparticles were dispersed in the monomer (OEOMA_500_) and the solvents anisole (1:1 v/v) and DMF (0.1:1 v/v). CuBr_2_ (200 ppm compared to the monomer) and Me_6_TREN ([Cu]/[ligand] = 1/3) were added. The reaction mixture was added to a clean, dry Schlenk flask. The reaction mixture was deoxygenated by sparging with nitrogen gas for 20 min, and the flask was placed in an oil bath. A reducing agent, Sn­(EH)_2_ ([Cu]/[Tin] = 1/5), was added to initiate ATRP. The reaction mixture was stirred at 50 °C until it became viscous. The HNP sample was purified via precipitation in hexane, followed by further dialysis in MeOH (1 cycle) and THF (2 cycles).

### Analysis

For gel permeation chromatography (GPC), samples were prepared by etching the silica nanoparticles with HF for 12 h and subsequently neutralizing them with ammonia. THF was used as the eluent with a flow rate of 1.00 mL/min at 35 °C in an Agilent GPC using Polymer Standards Services (PSS) columns (guard, 10^5^, 10^3^ and 10^2^ Å) and a differential refractive index detector (Waters, 2410) to determine number-average molecular weight (*M*_n_) and molecular weight dispersity (*M*_w_/*M*_n_).

Scanning electron microscopy (SEM) images were collected by using a Quanta 600 FEG instrument.

Thermogravimetric analysis (TGA) was carried out using a TA Instruments 2950 to determine the inorganic fraction of the HNPs. The data were analyzed using TA Universal Analysis. The heating procedure included three steps: ramping up at a rate of 30 °C/min to 120 °C, holding at 120 °C for 10 min, and ramping up at a rate of 20 °C/min to 800 °C. The inorganic fraction was calculated from the weight loss between 120 and 800 °C.

Grafting density (GD) was determined using eq 1

1 $$\mathrm{GD}=\frac{\left(1-f_{\mathrm{inorg}}\right)N_{A}\rho d}{6f_{\mathrm{inorg}}M_{n}}$$

*f*_inorg_: inorganic fraction determined via TGA; *N*_A_: Avogadro’s constant; ρ: density of nanoparticle material; d: diameter of nanoparticles; *M*_n_: molecular mass of polymer chains determined via GPC.

Mechanical testing was performed by using an Anton Paar MCR-302 Rheometer fitted with a parallel plate tool. HNPs were drop-cast onto the plate, and a nominal force of 1 N was applied. At room temperature, frequency sweeps were carried out from 0.01 to 100 s^–1^ to determine the storage and loss moduli. Creep tests were performed by applying a shear stress of 50 Pa for 300 s and 0 Pa for 600 s consecutively.

Electrochemical testing was carried out in CR2032-type coin cells at room temperature. All cells were prepared in a glovebox with water and oxygen contents of less than 0.5 ppm. The cathode material was produced through a slurry consisting of 85 wt % of commercial LFP powder, 10 wt % of Super-P, 5 wt % of poly­(vinylidene difluoride) binder, and NMP as the solvent. The mixture was cast on aluminum foil with a mass loading of 1.5 mg cm^–2^. The liquid electrolyte was prepared as a 1 M LiTFSI solution in DOL and DME (1:1 v/v) with 2 wt % LiNO_3_. During cell assembly, 60 μL of liquid electrolyte was used.

For ionic conductivity measurements, the HNP material was sandwiched in stainless steel|HNP mixed with LiTFSI (EO:Li = 10:1)|stainless steel cells. AC impedance spectroscopy was carried out at 25, 30, 40, 5, and 60 °C. Before each measurement, the cells were equilibrated for 15 min at each temperature. Using the charge-transfer resistance as well as the following equation, ionic conductivity was determined.

2 $$\sigma =\frac{L}{R\times A}$$

*L*: thickness of the HNP material; *A*: area of the material; *R*: charge-transfer resistance determined from the Nyquist plots

For the aSEI, the HNP sample (5 mg) was drop-cast onto lithium chips (13 mm diameter and 0.6 mm thickness) with a mass loading of 1.66 mg cm^–2^. After drying for 3 h at 65 °C, the HNP@Li anode was assembled with LFP as the cathode material.

The HNP sample was spray-coated on the separator material with the same mass loading that was applied for the aSEI. After drying in a vacuum oven at 80 °C for 12 h, this separator was used to assemble coin cells with the coated side facing the lithium anode.

For the liquid electrolyte additive, the HNP sample (5 mg) was dispersed in 60 μL of liquid electrolyte used for one coin cell. This mixture was then further used to assemble the batteries.

Rate capability measurements were carried out at 0.1, 0.2, 0.5, 1, and 2 C-rates for 5 cycles each to determine the specific discharge capacities. After the completion of 6 formation cycles at a 0.1 C-rate, the long-term cycling was performed at a C-rate of 0.5 for 550 cycles.

Symmetric Li|Li cells were assembled by coating the lithium chips according to the aSEI procedure. Consecutively, the cell was cycled at 1 mA cm^–2^ for 30 min per charge as well as discharge cycle.

Li|Cu cells were assembled with one lithium chip and a copper foil as the respective electrodes. The copper foil was loaded with lithium via charging the cell at 0.1 mA cm^–2^ for 10 h. Following this, the charge and discharge cycles were carried out at 1 mA cm^–2^ for 30 min.

## Results and Discussion

Hairy nanoparticles (HNPs) were synthesized by grafting oligo­(ethylene oxide) methyl ether methacrylate (OEOMA_500_) via activator regenerated by electron transfer atom transfer radical polymerization (ARGET ATRP) from SiO_2_ nanoparticles functionalized using 3-(chlorodimethylsilyl)­propyl α-bromoisobutyrate (Figure 2a).^28,29^ Molecular properties of the synthesized materials are presented in Table 1. High grafting densities of 0.49–0.59 chains/nm^2^ and varying molecular masses from 64,700 to 297,000 g mol^–1^ resulted in materials with high polymer content and low values of the inorganic fraction of 2.3–8.4%.

**Table 1:** Molecular Properties of Synthesized SiO_2_-g-OEOMA_500_ Hairy Nanoparticle Samples

Sample	*M*_n,app_ ^a^	*M*_n,abs_ ^b^	*Đ*	*f*_inorg_ ^c^	GD_abs_ ^d^
1	52,200	64,700	1.42	8.4	0.59
2	111,400	162,000	1.58	4.2	0.49
3	183,700	297,000	1.49	2.3	0.49

a Determined via gel permation chromatography in DMF at 50 °C, calibrated using poly­(methyl methacrylate) standards.

b Calculated with Mark–Houwink parameters determined in a previous report.^27^

c Determined via thermogravimetric analysis.

d Calculated by eq 1 using *M*_n,abs_.

**Figure 2:**
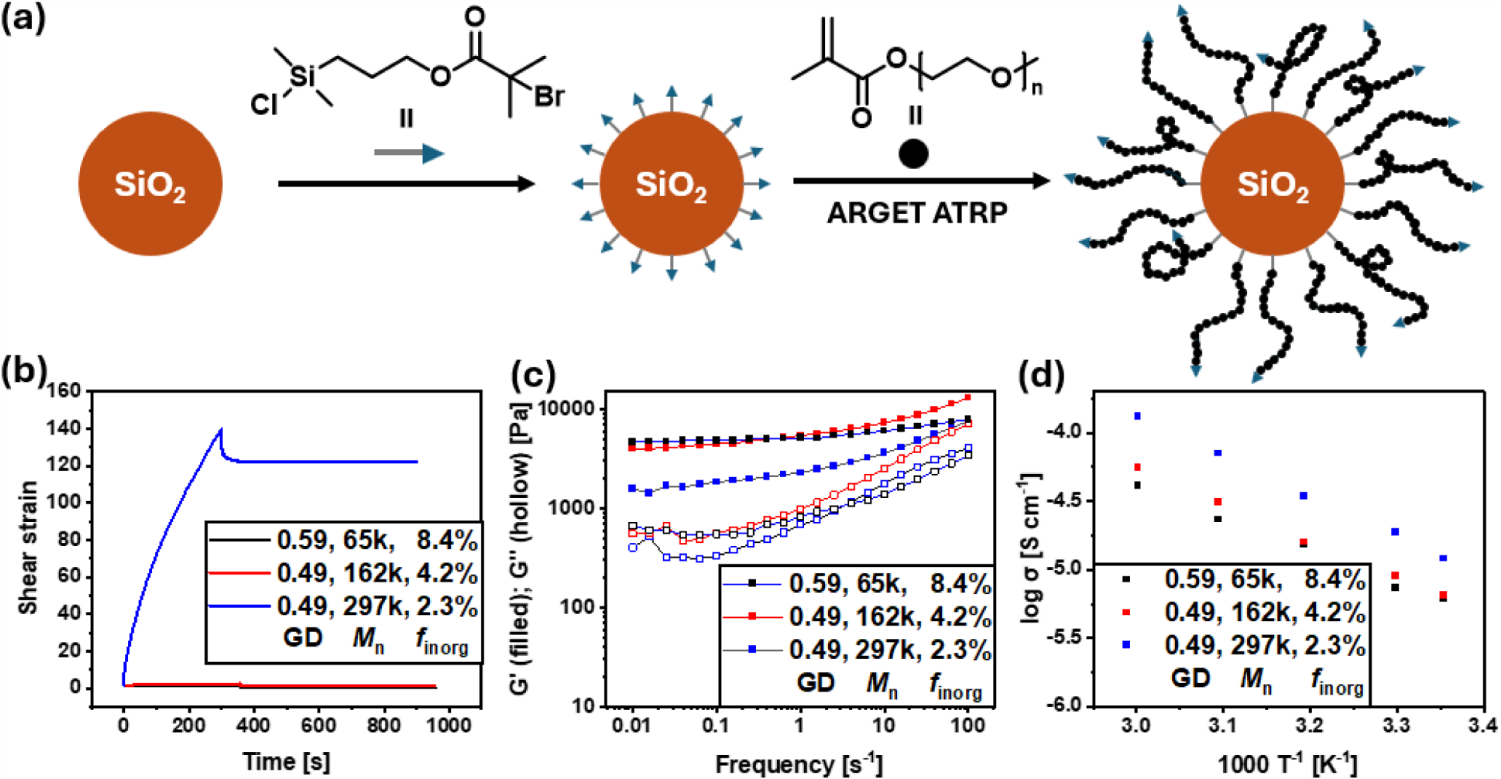
Schematic illustration of SiO_2_ NP surface functionalization and subsequent surface-initiated polymerization (a), creep test (b), frequency sweep (c), and ionic conductivity of HNP materials (d).

The mechanical properties of the HNP materials were tested through a creep test at a shear stress of 50 Pa and a frequency sweep at ambient temperature from 0.01 to 100 s^–1^ using a dynamic mechanical analyzer (DMA) (Figure 2b,c). While the samples with low and intermediate molecular mass showed low shear strains of 1.33 and 2.56%, grafting high molecular mass polymers of 297,000 g mol^–1^ led to a highly elastic material with a shear strain of 140% (Figure 2b). This was attributed to the longer polymer chains, which exceeded the concentrated polymer brush regime close to the nanoparticle surface, where they are highly extended.^30^ In the semidilute polymer brush regime, the polymer chains exhibited improved chain mobility. Hence, they were able to form interparticle entanglements, leading to the significantly increased shear strain of the material. The frequency sweep showed similar storage moduli for the low and intermediate molecular mass samples (Figure 2c).

At low frequencies, the decreasing values of storage moduli follow the order of the molecular mass. Normalized storage moduli at the lowest frequency were plotted against the molecular mass of all samples (Figure S1a). A linear trend was observed since the relaxation time is below the applied frequency. This trend can be ascribed to the combined effect of the molecular weight and inorganic fraction. Since molecular mass and inorganic fraction are correlated through eq. S1, their effects cannot be separated for similar grafting densities. To further reveal the impact of graft architecture on the structure and dynamics of brush particle films, the tan delta vs frequency curves at ambient temperature were plotted (Figure S1b). Interestingly, the reduction of the inorganic fraction resulted in the slowdown of the characteristic relaxation process, which is assumed to be the α-relaxation of the polymer chains. This supports the hypothesized formation of entanglements in the high molecular mass case, which is expected to limit mobility of the grafted chains.

Ionic conductivity was determined by mixing the HNPs with lithium bis­(trifluoromethane) sulfonimide (LiTFSI) in a ratio of [EO]:[Li] = 10:1. Herein, decreasing values with an increasing inorganic fraction were observed (Figure 2d). This is attributed to the increasing amount of nonconductive silica nanoparticles. Hence, the material with the highest molecular mass polymers showed the highest ionic conductivity of 0.015 mS cm^–1^.

The main challenge with ex-situ applied solid polymer electrolytes and other protective layers is the lack of interfacial contact between the anode and polymer. Therefore, lithium deposition was significantly hampered and could occur only in spots where lithium came in contact with the material. With sufficient elasticity, the material could fully encase the lithium anode surface. During cycling, together with the required mechanical strength, this resulted in uniform lithium deposition. Therefore, the combination of high elasticity while maintaining mechanical strength and high ionic conductivity is crucial for ex-situ protective layers in lithium–metal batteries. Consequently, the material with the highest molecular mass of polymer chains was chosen as a promising candidate for further testing. The stability of this HNP material was demonstrated through stable long-term cycling in a symmetric HNP@Li|HNP@Li cell (Figure S2).

To study the effect of the HNPs applied at different areas of the lithium–metal coin cell, three application techniques were carried out. First, the HNP sample was directly drop-cast onto the lithium anode prior to cell assembly as an artificial solid electrolyte interface (aSEI). Additionally, the HNPs were deposited on the separator via spray coating. Finally, as a control, the HNPs were dispersed in the liquid electrolyte to analyze the effect of the simple presence of HNPs without the formation of a physical solid layer.

Each of these application techniques was initially tested in Li|Cu cells (Figure S3). Bare lithium showed asymmetric cycling within the first few cycles. Additionally, HNPs applied as an aSEI or in the liquid electrolyte show increased voltages and asymmetric cycling at low cycle numbers, respectively. HNPs applied as the separator coating, however, led to symmetric cycling for the largest number of cycles, indicating stable stripping/deposition behavior. It should be noted that the limited performance at higher cycle numbers is attributed to poor interfacial contact, which hinders lithium deposition on the copper foil.

The HNP material was applied in Li|lithium iron phosphate (LFP) coin cells, which were tested at different current densities of 0.1, 0.2, 0.5, 1, and 2 C-rate for each 5 cycles (Figure 3a). At low current densities of 0.1–0.5 C-rate, all cells showed similar behavior with specific discharge capacities ranging from 125 mAh g^–1^ to 165 mAh g^–1^. At a 1 C-rate, the separator coating and bare lithium showed slightly lower discharge capacities of ∼115 mAh g^–1^ compared to ∼125 mAh g^–1^ for the aSEI and liquid electrolyte additive (LEA). Bare lithium anode and LEA showed the highest discharge capacities at 2 C-rate, with 101 mAh g^–1^ and 102 mAh g^–1^. Cells with an aSEI and separator coating exhibited a significant decrease, with values of ∼ 77 mAh g^–1^ and ∼66 mAh g^–1^, respectively. This was ascribed to additional solid layers in the cells with aSEIs and separator coatings. The pure HNP material shows lower ionic conductivity compared to liquid electrolytes. Hence, applying the HNPs as a solid layer, either as an aSEI or as a separator coating, led to a local decrease in ionic conductivity. At low current densities, this difference did not affect the performance due to the slow charge/discharge rates. However, higher current densities of >2 C-rate led to fewer lithium ions being transported to the electrode, resulting in diminished capacity. Dispersing the HNPs in the liquid electrolyte did not significantly impact lithium transport. Therefore, no change in performance was observed compared to the liquid electrolyte system. All samples showed good reversibility when subsequently charged at 0.1 C-rate, indicating the stability of the lithium-polymer interface.

**Figure 3:**
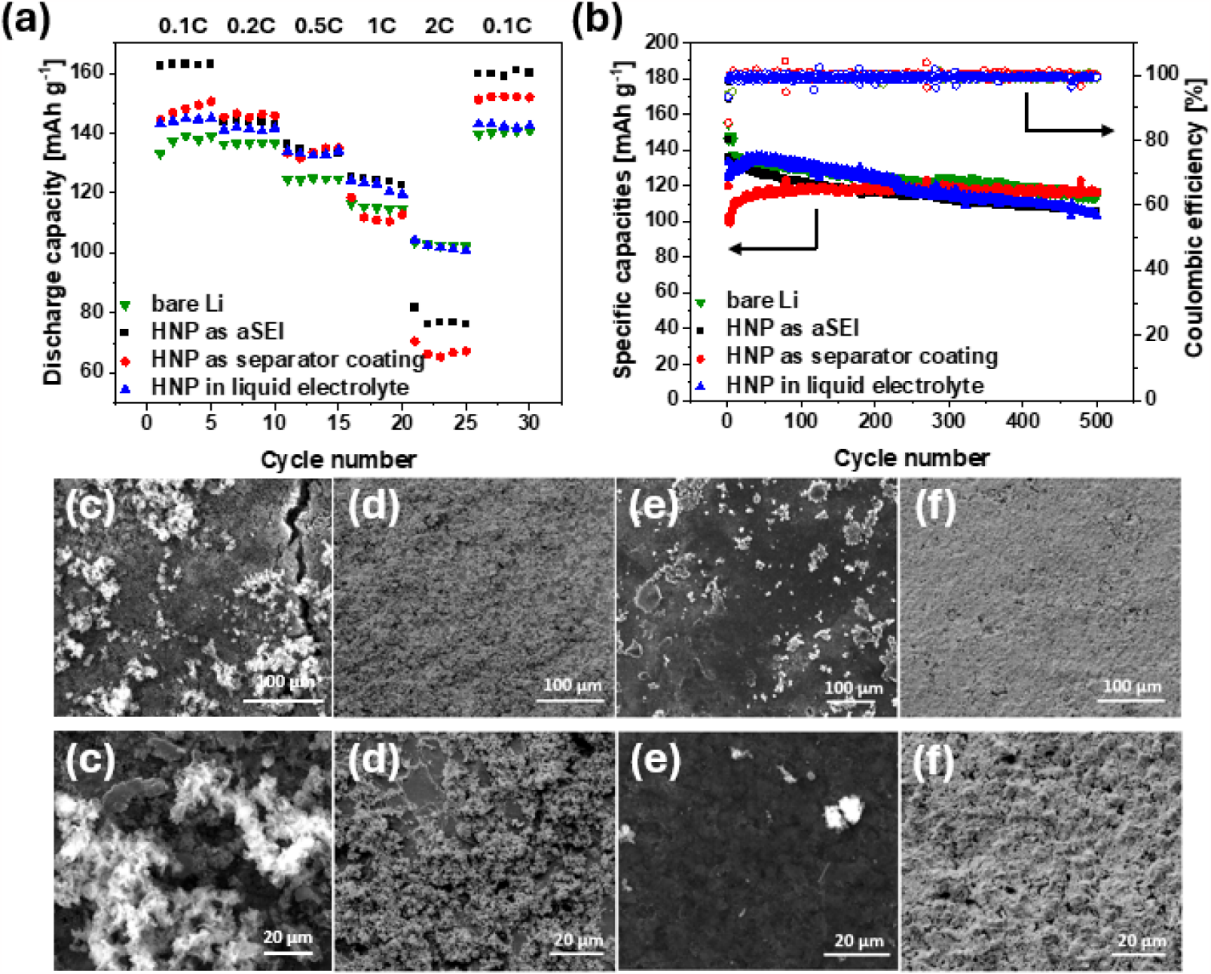
Rate capability test (a) and long-term cycling (b) in Li|LFP cells of HNP as aSEI, separator coating, and liquid electrolyte additive; SEM images of lithium metal anodes unprotected (c) and protected by HNP as liquid electrolyte additive (d), aSEI (e), and separator coating (f) after 500 cycles.

Furthermore, after five cycles at 0.1 C-rate, Li|LFP full cells were cycled at 0.5 C-rate for 500 cycles (Figure 3b). Using an unprotected lithium anode, the discharge capacities showed a constant decrease, leading to a capacity retention of 75% after cycling. With the understanding that cells break in during the first 75 cycles, the capacity retentions were calculated with the highest recorded capacity during that time. The cell with HNPs as LEA showed high discharge capacity during the initial cycles; however, the discharge capacity showed a constant decrease after ∼25 cycles. A discharge capacity of 77% indicated the low uniformity of lithium deposition with dispersed HNPs. When the HNPs were applied as an aSEI, the discharge capacity during the cycling decreased from 145.7 mAh g^–1^ to 106.1 mAh g^–1^. A capacity retention of 73% indicated significant drawbacks to using this HNP application method. With a separator coating, the discharge capacity increased from 101.1 mAh g^–1^ to 115.9 mAh g^–1^. The small effect was ascribed to stabilization of the polymer-lithium interface. After 75 cycles, the battery cell using the HNP material as a separator coating showed constant discharge capacity of ∼116 mAh g^–1^ leading to a capacity retention of 86% after 500 cycles. These results show improved results compared to those of other inorganic and organic separator coatings (Table 2).

**Table 2:** Comparative Analysis of Various Separator Coatings for Lithium–Metal Batteries (PP: Polypropylene; EAA: Poly­(ethylene-co-acrylic Acid))

Refs	Separator/coating	Rate	Cycle number	Capacity retention [%]
This work	PP/HNP	0.5 C	500	86
 ^31^	PP/polymer	0.2 C	100	92
 ^32^	PP/polymer + silica aerogel	1 C	100	81
 ^33^	PP/polymer + modified SiO_2_ NPs	1 C	300	95
 ^34^	PP/black phosphorus	1 C	400	79
 ^35^	EAA/SnO_2_ nanospheres	1 C	1000	55

To compare the ability of the protective layer to provide uniform lithium deposition, the surface morphology of the anode was studied via scanning electron microscopy (SEM). For the bare lithium anode, the formation of nonuniform growths was observed (Figure 3c) and ascribed to the expected nonuniform lithium deposition during the cycling process. The addition of the HNPs to the liquid electrolyte led to lithium being deposited in a similarly nonuniform manner compared to bare lithium (Figure 3d). Next to these growths, smooth surfaces were observed, which indicated that no new lithium was deposited in these areas. Additionally, the formation of cracks was observed. While the HNP material as an aSEI provided a smooth anode morphology, the cell in which an aSEI was utilized produced a higher amount of “dead” lithium (Figure 3e). This was potentially caused by the application method, as drop casting the HNP material resulted in small irregularities due to nonuniform solvent evaporation. To maximize ion transport, lithium would be deposited in areas with a thinner HNP coating. These nonuniform depositions then resulted in growths that could subsequently break off as “dead” lithium. The formation of this lithium species caused the previously observed decrease in capacity retention for the HNP as an aSEI. In contrast, the separator coating provided a lithium surface with minimal roughness (Figure 3f). While the protective layer did not result in completely uniform deposition, the anode surface showed a similar roughness across its area. This observation, in combination with high capacity retention, is consistent with a stable polymer-lithium interface, resulting in stable stripping and plating processes. This result can also be attributed to the application of the HNP material via spray coating, which facilitates a significantly smoother administration of the polymeric material.

## Conclusion

In this work, the application of hairy nanoparticles (HNPs) in different areas of lithium–metal batteries was studied. HNPs dispersed in the liquid electrolyte showed similar results compared to cells with no HNP addition. This was attributed to the lack of a protective coating in both cells, leading to nonuniform lithium deposition during cycling. HNPs applied as an artificial solid electrolyte interface (aSEI) caused irregularities due to the drop-casting of the HNP material, which led to an increase in the “dead” lithium formation. Spray-coating the material ex situ on the separator allowed for a smoother application of the protective layer. Additionally, the most common downfall of ex-situ applied separator coatings was inadequate interfacial contact between the protective coating and the anode. This was addressed by using a material with higher elasticity. An appealing capacity retention of 97% was achieved due to the smooth protective layer, which provided uniform lithium deposition. This allowed for shorter handling times of highly reactive lithium. Hence, safety during cell assembly was enhanced while maintaining high battery performance.

## Supplementary Material



## Notes

Long-term symmetric cycling; Li|Cu half cell cycling (PDF)
