# Supplementary material for: Elastic Hairy Nanoparticle Separator Coating for Enhanced Interfacial Stability in Lithium–Metal Batteries
Source: ACS Appl Polym Mater. 2025 Sep 24;7(19):13219–25. doi: 10.1021/acsapm.5c02697 (PMC12519443; doi:10.1021/acsapm.5c02697)
Supplement: Supplementary file 1 [file ap5c02697_si_001.pdf]

Supporting Information: Elastic Hairy Nanoparticle Separator Coating for Enhanced Interfacial Stability in Lithium-Metal Batteries

Verena Kempkes[a], Sipei Li[a], Jay F. Whitacre*[b], Krzysztof Matyjaszewski*[a]

[a]Department of Chemistry

Carnegie Mellon University

4400 Fifth Avenue, Pittsburgh, PA 15213, USA

E-mail: matyjaszewski@cmu.edu

[b]Department of Materials Science and Engineering

Carnegie Mellon University

5000 Forbes Avenue, Pittsburgh, PA 15213, USA

E-mail: whitacre@andrew.cmu.edu

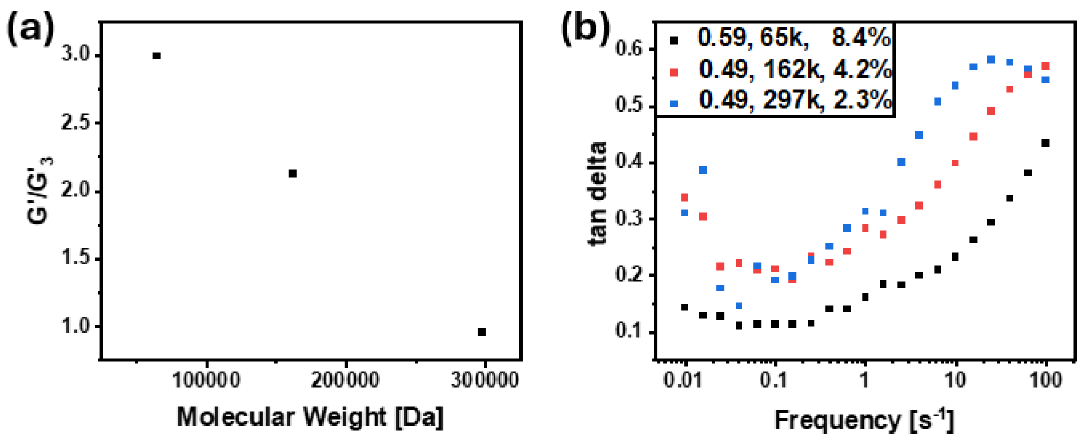

Figure S1: Relationship of G' to molecular mass at 0.01 s^{-1} (a) and tan delta curves for frequency sweeps (b).

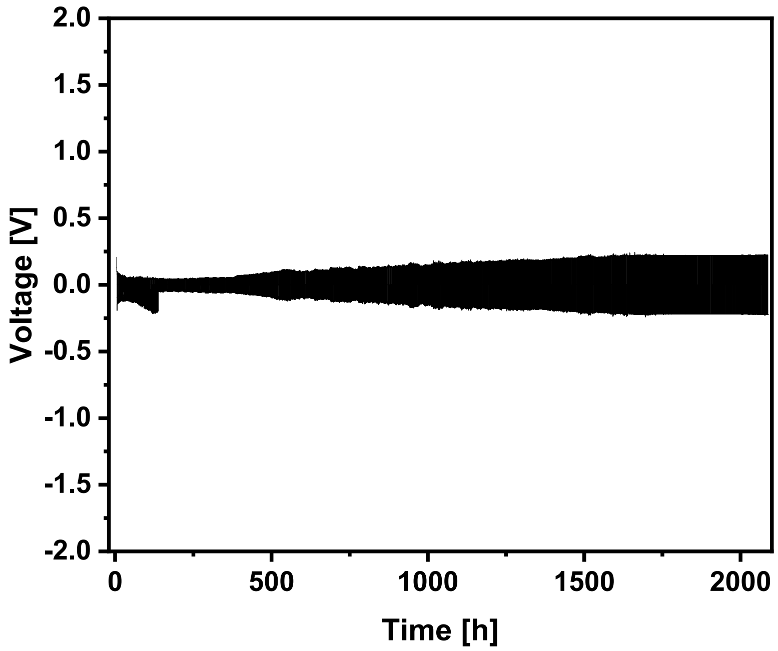

Figure S2: Symmetric cycling of Sample 3 in HNP@Li|HNP@Li cell at 1 mAh cm^{-2}.

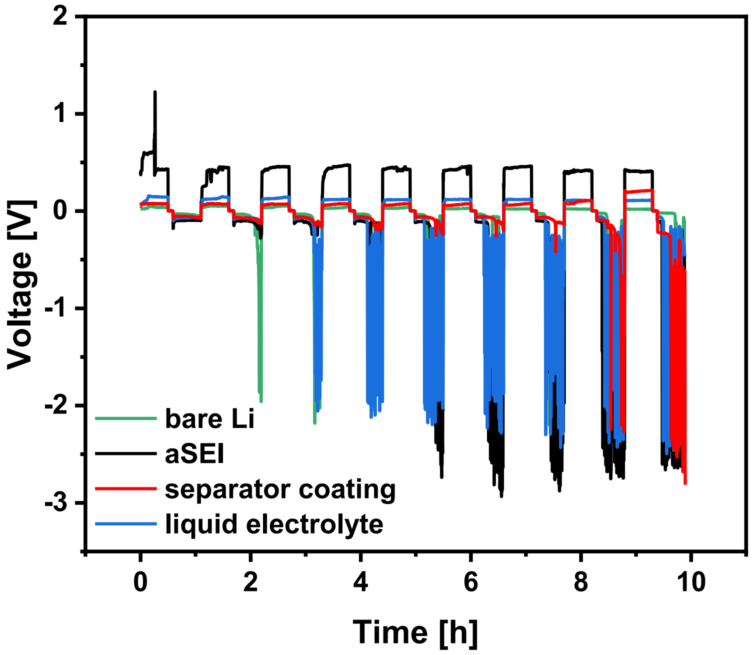

Figure S3: Cycling in Li|Cu cells at 1mAh cm^{-2}.
